# Pre-frailty and frailty of elderly residents in a municipality with a low Human Development Index

**DOI:** 10.1590/0104-1169.3538.2464

**Published:** 2014

**Authors:** Wanderley Matos Reis, José Ailton Oliveira Carneiro, Raildo da Silva Coqueiro, Kleyton Trindade Santos, Marcos Henrique Fernandes

**Affiliations:** 1 MSc, Assistant Professor, Departamento de Saúde, Universidade Estadual do Sudoeste da Bahia, Jequié, BA, Brazil; 2 PhD, Adjunct Professor, Departamento de Saúde, Universidade Estadual do Sudoeste da Bahia, Jequié, BA, Brazil; 3 Doctoral student, Universidade Federal da Bahia, Salvador, BA, Brazil. Assistant Professor, Universidade Estadual do Sudoeste da Bahia, Jequié, BA, Brazil; 4 Master's student, Universidade Estadual do Sudoeste da Bahia, Jequié, BA, Brazil

**Keywords:** Aging, Frail Elderly, Health Status

## Abstract

**OBJECTIVE:**

to identify the prevalence of the factors associated with pre-frailty and frailty of elderly residents in a municipality with a low Human Development Index

**METHOD:**

Cross-sectional study with a populational and household framework conducted with 316 elderly people. Frailty was determined from the presence of three or more of the following factors: (i) self-reported unintentional weight loss; (ii) lack of strength and energy; (iii) weakness; (iv) slowness; (v) low level of physical activity. The association between frailty and socio-demographic, behavioral and health factors was measured using the multinomial logistic regression technique.

**RESULTS:**

The prevalence of pre-frailty and frailty was 58.7% and 23.8%, respectively. The adjusted regression model showed that the state of pre-frailty was associated with gender, age group and BMI, and frailty was associated with gender, age group, hospitalization, functional capacity, and self-perceived health.

**CONCLUSION:**

The evidence presented in this study demonstrates more variables associated with the frailty condition, reinforcing the concept of a multifactorial clinical syndrome that may result in the loss of functionality.

## Introduction

The aging process brings changes, which together with the increased prevalence of chronic diseases can cause the onset of geriatric syndromes, among which the frailty syndrome is highlighted. Frailty includes factors of different orders, being characterized as a syndrome resulting from the loss of physiological reserves and adaptation to stressors, where the energy deficit, sarcopenia, decreases the muscle strength and tolerance to effort leading to an exacerbated decline in multiple systems that places the individual in a condition of greater vulnerability^(^ 1 ^)^. 

Frailty has increasingly emerged as an important concept, both in the clinical care of older people, as well as in studies on aging. As a clinical syndrome, it is usually associated with an increased risk of adverse situations such as falls, disabilities, institutionalization, and death^(^ 2 ^)^. There is no defined scientific consensus regarding the term frailty, its definition and its indicators, nor how it can be identified or even evaluated. However, the majority of studies that deal with frailty generally define it as an unstable condition related to functional decline, from the interaction of the individual with the environment, in which, an event considered of minor impact can cause limitations in the performance of voluntary activities and result in the loss of autonomy and functional capacity^(^ 3 ^)^.

The reversal of the installation of advanced frailty frameworks may be linked to the identification of factors that are considered open to modifications, such as socioeconomic conditions, lifestyle, and social support, in which the early identification of signs and symptoms causing the frailty syndrome may indicate the adoption of objective interventions that prevent complications of the frailty and injuries in the elderly population^(^ 4 ^)^.

In Brazil, the majority of municipalities experience an increase in the number of elderly in the population each year. They present poor health indicators and this may contribute to the development of early frailty resulting in an unfavorable prognosis with dependence, hospitalization and severe complications in the subsequent years^(^ 4 ^)^. In addition, research into the identification of frailty in the elderly is at an early stage, therefore there are few population-based studies that present factors involved in the process of frailty in the Brazilian population.

When studying frailty it is essential to consider the context in which this process takes place. Since Brazil is a country of continental dimensions it is essential to understand how frailty develops in regions marked by pronounced social inequalities. Thus, the aim of this study was to identify the prevalence and the factors associated with pre-frailty and frailty of elderly residents in a community with a low human development index.

## Method

This is a cross-sectional study that is part of epidemiological study with a community and populational basis, the title of which is: Nutritional status, risk behaviors and health conditions of the elderly people of Lafaiete Coutinho-BA. A census was conducted in January 2011 with elderly people residing in urban areas, registered in the Family Health Strategy, which achieves 100% coverage. All those aged ≥60 years (n=355) were contacted for interviews and to carry out examinations (blood tests, arterial pressure measurement, anthropometric measures, and motor tests). Of the 355 elderly people, 316 participated in the study (89.0%). There were 17 refusals (4.8%) and 22 (6.2%) individuals were not located in their residences after three visits at different times.

A specific form was used, based on the questionnaire used in the Health, Welfare and Aging Study - SABE (http://hygeia.fsp.usp.br/sabe/quetionario.php), conducted in seven countries in Latin America and the Caribbean^(^ 5 ^)^, with the International Physical Activity Questionnaire (IPAQ)^(^ 6 ^)^, adapted for the elderly^(7) ^added to this, as well as the Geriatric Depression Scale (GDS), used for screening for depression in the elderly and consisting of 15 questions with yes or no answers, also having been validated for use in Brazil^(^ 8 ^)^.

Frailty (dependent variable) was identified according to the modified version of frailty, considering five components^(^ 1 ^)^: 1. Weight Loss: defined by the self-report of unintentional weight loss of ≥3 kg during the 12 months preceding the study^(^ 9 ^)^, as the instrument used was based on the SABE study, which does not make an objective prediction for weight loss. 2. Muscle weakness: Manual Gripping Force (MGF) was evaluated by means of a hydraulic dynamometer (Saehan Corporation SH5001, Korea). Frailty was defined according to the gender and body mass index (BMI). For each category, the cutoff points for the MGF (Kgf) were fixed in the 25th percentile, with adjustments for gender and BMI. The cutoff points for men adopted were: 0<BMI<22 - MGF≤19Kgf.; 22≤BMI≤27 - MGF≤21Kgf., BMI>27 - MGF≤22Kgf.; and for women: 0<BMI<22 - MGF≤11Kgf.; 22≤BMI<27 - MGF≤15Kgf., BMI>27 - MGF≤14Kgf. 3. Low resistance and energy: defined based on two questions of the GDS^(^ 8 ^)^: "Have you put aside many of your activities and interests?" and "Do you feel full of energy?". A positive answer to the first question and/or a negative answer to the second were considered evidence of low resistance/lack of energy. 4. Slowness in the walking test: defined through the physical performance of a 2.44m walking test. The slowness was adjusted according to the gender and height of the elderly person. The height was divided into two categories based on the median (50^th^ percentile) men ≤1.61m and women ≤1.49m, below or equal to the median; men >1.61m and women >1.49m, above the median. For each category the cutoff points that considered the individual slow in the walking test were fixed in the 75^th^ percentile: below or equal to the median, ≥5s and ≥6s (for men and women, respectively); above the median, ≥4s (for both genders). Low level of physical activity: The instrument used to evaluate the level of habitual physical activity was the International Physical Activity Questionnaire (IPAQ)^(^ 7 ^)^. The individuals who performed less than 150 minutes per week of moderate and/or vigorous physical activity were considered insufficiently active.

An ordinal variable with scores ranging from zero to five (0-5) was created from the sum of the points of all the components, with the following classification adopted: 0 points = not frail; 1-2 points = pre-frail; ≥3 points = frail^(^ 1 ^)^. All the individuals who responded to only 3 components and that were classified as frail were considered. Individuals who answered at least 4 components for the classification of frailty were considered eligible for the other classifications^(^ 9 ^)^. Thus, 286 subjects, classified according to frailty phenotype, were included in the analysis.

The explanatory variables were: 1. Sociodemographic: Gender, Age group, Knowledge of how to read and write a message, Family arrangement, Participation in religious activity. 2. Behavioral: Consumption of alcoholic beverage, and Use of tobacco. 3. Health conditions: Hospitalization in the previous year, Body Mass Index BMI: (BMI<22kg/m^2^ = underweight, 22 kg/m^2^≤BMI≤27kg/m^2^ = appropriate, and BMI>27kg/m^2^ = overweight)^(^ 10 ^)^, Fall event in the previous year, Number of self-reported chronic diseases, and Functional capacity (measured through the Basic Activities of Daily Living (BADL) using the Katz scale^(^ 11 ^)^ and Instrumental Activities of Daily Living (IADL), using the Lawton scale^(^ 12 ^))^. 

 Amount of medications used, Self-perceived health, and Cognitive status (assessed through the Mini-Mental State Examination questionnaire. using the validated a modified version^(^ 13 ^)^ to verify the reliability of the answers - score >12 = not compromised and score ≤12 = compromised). When the cognitive status score was not reached, the informant was asked to responded to another instrument, which was the Functional Activities questionnaire of Pfeffer^(^ 14 ^)^, with information pertaining to the elderly person, thus evaluating the need for a substitute informant during the interview.

The associations between frailty and the explanatory variables were verified by obtaining crude and adjusted estimates of the odds ratio, using a confidence interval of 95%, through a multinomial logistic regression model. Variables that showed statistical significance of at least 20% (p≤0.20) in the crude analysis were included in the adjusted analysis, following the order of a hierarchical model for the determination of the outcomes^(^ 15 ^)^.

According to the model established, the variables of the upper levels (distal) interact among themselves and determine the variables of the lower levels (proximal). Regarding the effect of each explanatory variable on the outcome, the variables of the same level and upper levels in the model were controlled for, with the statistical criterion for remaining in the model being 20% (p≤0.20).

The study was approved by the Research Ethics Committee of the State University of Southwest Bahia (nº 064/2010). Data were tabulated and analyzed using the Statistical Package for the Social Sciences for Windows (SPSS^(r))^, version 16.0 program.

## Results

The prevalence of pre-frailty and frailty of the elderly people living in the urban area of the municipality of Lafaiete Coutinho-BA was 57.8 and 23.8%, respectively.

Table 1 shows the crude analysis of the independent variables that composed the socio-demographic, behavioral, and health condition factors and their association with frailty in elderly people.

**Table 1:** Association between sociodemographic, behavioral, and health condition factors and pre-frailty and frailty in elderly people living in the community. Lafaiete Coutinho, BA, Brazil, 2011

Variables		Frail				Pre-frail			*p*-value
		n	%	OR_crude_ (CI 95%)		n	%	OR_crude _(CI 95%)	
Gender									0.002
	Male	31	23.7	1		66	50.4	1	
	Female	37	23.9	2.53(1.18-5.43)		102	65.8	3.28(1.68-6.41)	
Age group									<0.001
	60-69 years	16	14.7	1		66	60.6	1	
	70-79 years	19	19.0	1.78(0.72-4.35)		63	63.0	1.43(0.71-2.85)	
	≥80 years	33	42.9	11.13(3.61-34.32)		39	50.6	3.19(1.13-8.96)	
Knows how to read and write a message				pas					0.029
	Yes	18	18.0	1		57	57.0	1	
	No	50	26.9	2.77(1.28-6.01)		111	59.7	1.94(1.02-3.69)	
Family arrangement									0.450
	Accompanied	54	22.6	1		141	59.0	1	
	Alone	14	29.8	1.90(0.67-5.35)		27	57.4	1.40(0.54-3.62)	
Religious activity									0.952
	Participants	63	23.7	1		156	58.6	1	
	Does not participate	5	25	1.24(0.28-5.46)		12	60	1.20(0.32-4.45)	
Alcohol consumption									0.329
	Does not consume	64	24.7	1		152	58.7	1	
	Consumes	4	14.8	0.38(0.10-1.39)		16	59.3	0.64(0.25-1.67)	
Use of tobacco									0.249
	Never smoked	20	17.2	1		73	62.9	1	
	Ex-smoker	39	28.5	1.95(0.88-4.29)		75	54.7	1.02(0.53-1.99)	
	Currently smokes	9	27.3	2.58(0.69-9.70)		20	60.6	1.57(0.48-5.08)	
Hospitalization in the previous year									0.003
	No	41	19.0	1		132	61.1	1	
	One or more times	27	38.6	4.04(1.58-10.30)		36	51.4	1.67(0.69-4.03)	
Body Mass Index									0.006
	Appropriate	34	26.8	1		64	50.4	1	
	Overweight	10	12.5	0.65(0.25-1.71)		57	71.3	1.98(0.94-418)	
	Underweight	22	28.9	2.34(0.90-6.05)		46	60.5	2.60(1.09-6.21)	
Fall Event									0.012
	No	41	19.2	1		132	61.7	1	
	Yes	26	36.6	2.88(1.20-6.91)		36	50.7	1.24(0.55-2.79)	
Number of chronic diseases									0.044
	None	13	22.4	1		30	51.7	1	
	One	17	17.5	1.03(0.38-2.77)		61	62.9	1.60(0.71-3.59)	
	Two or more	37	31.1	3.04(1.16-7.99)		68	57.1	2.42(1.04-5.65)	
Functional capacity									< 0.001
	Independent	12	9.5	1		79	62.7	1	
	Dependent in the Instrumental Activities of Daily Living (IADL)	37	31.4	9.81(3.83-25.10)		70	59.3	2.81(1.33-5.96)	
	Dependent in the Basic Activities of Daily Living (BADL) and Instrumental Activities of Daily Living (IADL)	16	43.2	15.55(3.84-62.86)		18	48.6	2.65(0.73-9.61)	
Use of medication									0.002
	Up to one	18	15.9	1		66	58.4	1	
	Two or more	50	28.9	3.83(1.76-8.35)		102	59.0	2.13(1.12-4.05)	
Self-perceived health									0.005
	Positive	18	15.7	1		68	59.1	1	
	Negative	46	27.7	3.52(1.61-7.71)		99	59.6	2.01(1.05-3.81)	
Cognitive State									0.004
	Uncompromised	32	17.4	1		111	60.3	1	
	Compromised	29	31.2	4.12(1.71-9.94)		55	59.1	2.25(1.02-4.97)	

A significant association was found in the crude analysis for the variables: female gender (p=0.002), age group (p<0.001), knows how to read and write a message (p =0.029), hospitalization in the previous year (p=0.003), BMI (p=0.006), fall event (p=0.012), number of chronic diseases (p=0.044), functional capacity (p<0.001), medication use (p=0.002), self-perceived health (p=0.005) and cognitive status (p= 0.004).

Table 2 presents the adjusted hierarchical analysis, through multinomial logistic regression, of the association between the independent variables that obtained significance, and the classification of the frailty phenotype.

**Table 2:** Final multiple multinomial logistic regression model of the association between pre-frailty and frailty and the independent variables of the study. Lafaiete Coutinho, BA, Brazil, 2011

Blocks	Variable		Frail	*p*-value	Pre-frail	*p*-value
			OR_Adjusted_ (CI 95%)		OR_Adjusted_ (CI 95%)	
A	Gender					
		Male	1		1	
		Female	2.39(1.08-5.28)	0.031	3.21(1.63-6.31)	0.001
	Age group					
		60-69 years	1		1	
		70-79 years	1.76(0.71-4.34)	0.216	1.41(0.69-2.86)	0.336
		≥80 years	10.75(3.46-33.39)	<0.001	3.04(1.06-8.68)	0.037
C	Hospitalization in the previous year					
		No	1		1	
		One or more times	3.19(1.10-9.27)	0.033	1.62(0.63-4.17)	0.313
	Body Mass Index					
		Appropriate	1		1	
		Overweight	0.50(0.15-1.63)	0.255	1.89(0.80-4.43)	0.143
		Underweight	2.40(0.77-7.42)	0.127	3.21(1.25-8.23)	0.015
	Fall Event					
		No	1		1	
		Yes	2.32(0.80-6.72)	0.120	0.90(0.36-2.20)	0.821
	Functional capacity					
		Independent	1		1	
		Dependent in the Instrumental Activities of Daily Living (IADL)	6.60(2.32-18.72)	<0.001	1.97(0.88-4.38)	0.097
		Dependent in the Basic Activities of Daily Living (BADL) and Instrumental Activities of Daily Living (IADL)	8.99(1.84-43.78)	0.007	1.63(0.41-6.50)	0.484
	Self-perceived health					
		Positive	1		1	
		Negative	3.49(1.40-8.69)	0.007	1.78(0.89-3.57)	0.102

In the first step the variables of the sociodemographic block were analyzed, and the variables that remained associated with the frail stage were: female gender (p=0.031) and age group ≥80 years (p<0.001) and, with the pre-frail stage, also female gender (p=0.037) and age group ≥80 years (p=0.001).

In the second step the variables of the health conditions block were included and those that continued to be associated with the frail classification were: hospitalization in the previous year, one or more times (p=0.033), functional capacity for both dependent in the IADL (p<0.001), as well as in the BADL and IADL (p=0.007), and also self-perceived negative health (p=0.007). Only the underweight BMI variable (p=0.015) remained associated with the pre-frail classification.

The variables related to behavioral aspects, i.e., consumption of alcoholic beverages and tobacco use were not included in the adjusted model, as they did not present at least 20% statistical significance (p≤0.20).

## Discussion

In the present study 23.8% of the elderly people were frail, 58.7% pre-frail, and 17.5% non-frail. The frailty prevalence values were higher when compared to international studies^(^ 16 ^)^. The explanation for the wide variation in the prevalence of frailty in the elderly is probably related to socioeconomic differences among the elderly people studied, as well as the methodological differences in the use of some instruments that differ from those that compose the items used^(^ 16 ^)^.

In the present study women had a higher prevalence in both the pre-frail and frail categories, corroborating other studies that indicated that elderly women are frailer than elderly men, and presented a frailty prevalence of over 13% for the men and 17% for the women. Over three years, the women had twice the pre-frailty incidence rates compared to the men^(^ 17 ^)^.

In a study conducted in Taiwan, which estimated the prevalence of frailty and identified factors associated with frailty, from the data of the Survey of Health and Living Status of the Elderly, it was found that the prevalence of frailty increased with age and was higher in women^(^ 18 ^)^.

Longevity is another aspect that should be taken into consideration. A national study, which is part of the multicentric project Frailty in Elderly Brazilians (*Fibra*), analyzed prevalence characteristics and associated factors related to frailty and, of the sociodemographic variables included in the model, only age was associated, even when adjusted for the other variables, thus demonstrating the influence of the aging process on the emergence of frailty^(^ 19 ^)^.

Weight loss has been reported to be important information related to the prediction of frailty. However, its participation in the phenomenon is still unclear, considering that weight loss does not precisely represent muscle loss, and a simultaneous increase in fat mass could mask the real estimate^(^ 20 ^)^. 

The studies that have investigated the association between the BMI and frailty are still inconclusive. Some have shown that pre-frail and frail elderly men presented lower BMI values and that the prevalence of frailty is slightly higher in normal and underweight men(20). Others, however, have reported an association of increased BMI values with the frail situation, mainly observed in frail elderly women^(^ 21 ^)^.

The findings of the present study were consistent with the results of the study in which five Latin American and Caribbean cities were investigated, including São Paulo, Brazil, which verified that higher BMI values for women were related to a greater probability of frailty^(^ 9 ^)^.

The observation of the association between dependence in performing instrumental and basic activities and frailty is presented in national and international studies, demonstrating the degree of injury that this impairment generates in the elderly, by directly limiting them in their autonomy, resulting in a decrease in the quality of life^(^ 1 ^)^. The data obtained are consistent with the literature in that, after adjustment, an association between the BADL, IADL and frailty was also found^(^ 18 ^)^.

However, it is critical to know how the process of functional disability takes place and its relationship to frailty, therefore important adverse factors are interrelated to the two events, such as fatigue, low levels of physical activity and decreased muscle strength, which have been suggested as predictors for functional disability^(^ 22 ^)^.

In this study, self-perceived health was associated with being frail, corroborating other findings^(^ 18 ^)^. There are suggestions in the literature that this issue is influenced by the life trajectory and the experiences of the elderly people and the way they deal with adverse situations^(^ 1 ^)^, and overcoming these situations has been sustained by the theory that proposes the relationship between resilience in the elderly and frailty^(^ 23 ^)^.

Some limitations of the study are that this research is characterized as a cross-sectional type study, in which there is no possibility of establishing cause and effect, and that some of the instruments used required subjective or self-reported information, which may lead to memory bias. Longitudinal studies and the use of more objective instruments are needed to make the inferences about the predictive indicators of frailty more robust.

## Conclusion

The prevalence of pre-frailty and frailty was 58.7% and 23.8%, respectively. The evidence presented in this study showed that the state of pre-frailty was associated with gender, age group and BMI. While the state of frailty was associated with gender, age group, hospitalization, functional capacity and self-perceived health. There were more variables associated with the frailty condition, which reinforces the concept of a multifactorial clinical syndrome that may result in the loss of functionality. Furthermore, it is worth mentioning once again that the high prevalence of elderly people in the pre-frail condition constitutes important information, considering the possibility of deterioration and becoming frail over a short period, which will result in more care and spending on the health of these elderly people.

Therefore, the results published on the subject should serve to guide and improve the healthcare policies for the elderly, as well as assist the professionals involved in their care, whose purpose should be to prevent deterioration and its consequences, and the development of the preliminary stages of frailty into an advanced situation.
